# Comparison of cognitive performance and aspects of the care context in elderly caregivers in Brazil: A follow-up study

**DOI:** 10.1590/1980-57642020dn14-020009

**Published:** 2020

**Authors:** Ana Carolina Ottaviani, Allan Gustavo Bregola, Mariélli Terassi, Bruna Moretti Luchesi, Érica Nestor Souza, Nathalia Alves de Oliveira, Francisco José Fraga, Sofia Cristina Iost Pavarini

**Affiliations:** 1 Federal University of São Carlos, Graduate Program in Nursing, São Carlos, SP, Brazil.; 2 School of Health Sciences, University of East Anglia, Norwich, United Kingdom.; 3 Central Paulista University Center, São Carlos, SP, Brazil.; 4 Federal University of Mato Grosso do Sul, Três Lagoas Campus, Graduate Program in Nursing, Três Lagoas, MS, Brazil.; 5 Federal University of ABC (UFABC), Engineering, Modeling and Applied Social Sciences Center (CECS), Santo André, SP, Brazil.

**Keywords:** aged, caregivers, cognition, mental health, primary health care, idosos, cuidadores, cognição, saúde mental, atenção primária à saúde

## Abstract

Cognitive health is important for the quality of life and well-being of elderly caregivers. Cognitive impairments can negatively affect the ability to care for oneself and others.

**Objective:**

To compare cognitive performance and aspects of the care context in elderly caregivers of older adults in a three-year follow-up investigation

**Methods:**

A longitudinal study was conducted of 61 elderly caregivers treated in primary care in a city in the interior of the state of São Paulo with data collected in 2014 and 2017. Sociodemographic characteristics, the care context, cognition, performance for activities of daily living, burden and depressive symptoms were collected in individual interviews. Data analysis was performed to compare categorical and continuous variables.

**Results:**

Significant increases were found between baseline and follow-up for the language domain score of the cognitive evaluation (p=0.024), receipt of material support (p=0.020), time providing care (p=0.045) and dependence of elderly care recipient for basic activities of daily living (p=0.042).

**Conclusion:**

Elderly caregivers performed better on the domain of language, received more material/financial support, spent more time on care and reported greater dependence of the elderly care recipient.

## RESUMO.

A saúde cognitiva é importante para a qualidade de vida e bem-estar dos cuidadores idosos. O comprometimento cognitivo pode afetar negativamente a capacidade de cuidar de si e dos outros.

**Objetivo:**

Comparar o desempenho cognitivo e os aspectos do contexto do cuidado em idosos cuidadores de idosos em uma de acompanhamento de três anos.

**Métodos:**

Trata-se de um estudo longitudinal com medidas em 2014 e 2017, realizado com 61 cuidadores idosos cadastrados na atenção primária de um município do interior do Estado de São Paulo. Características sociodemográficas, contexto do cuidado, cognição, desempenho nas atividades da vida diária, sobrecarga e sintomas depressivos foram coletados em entrevistas individuais. A análise dos dados foi realizada para comparar variáveis categóricas e contínuas.

**Resultados:**

Foram encontrados aumentos significativos entre a linha de base e o acompanhamento do escore no domínio da linguagem da avaliação cognitiva (p=0,024), receber ajuda material (p=0,020), tempo de cuidado (p=0,045) e dependência do idoso em relação às atividades básicas da vida diária (p=0,042).

**Conclusão:**

Os cuidadores de idosos tiveram melhor desempenho no campo da linguagem, passaram a receber mais apoio material / financeiro, experimentaram aumentos no tempo gasto no cuidado e na dependência do idoso.

Changes in the demographic and epidemiological profile of the population have resulted in a higher frequency of chronic conditions, which can compromise the functional capacity of elderly individuals, leading to the need for daily care.^1^ Culturally, care in Brazil occurs in the family environment. Brazilian caregivers are mostly women (wife or daughter of the care recipient), in a stable relationship (married or with a long-term partner), middle-aged (45 to 50 years), have a low level of education and provide care for more than ten hours a day^2^^-^ 4 Moreover, some studies show there is a growing number of people over 60 who play the role of informal caregiver of another more dependent elder.^5^^-^ 7

While the task of providing care can offer benefits and positive results, there is a high rate of excessive burden resulting from emotional stress, physical exhaustion and health problems, as well as limitations in terms of social and leisure activities.^8^^-^ 10 Providing care is a complex, challenging task that can exert an impact on the mental and physical health of the caregiver.^11^ As cognitive performance is important to the quality of life and wellbeing of elderly caregivers, cognitive decline can negatively affect their ability to provide care and even perform self-care.^12^ Studies have shown that elderly caregivers have poorer performance on tasks of cognitive processing, executive functions, attention and memory.^13^^-^ 15

Another issue that requires further evidence in studies on cognitive health, particularly among caregivers, relates to emotional health. High levels of stress resulting from the burden of providing daily care can jeopardize the cognitive health of caregivers^5^^,^ 15 ^,^ 16 A systematic review reported high levels of stress in family caregivers of elderly individuals with dementia, resulting in poorer performances with regard to attention and executive functions; however, interventions with the aim of reducing stress are reported to improve cognition.^17^ A study involving 252 elderly caregivers of older adults with dementia compared to demographically matched non-caregiver controls found that cognitive performance was similar in both groups, but the caregivers had higher levels of stress and depressive symptoms.^18^

The literature shows the importance of preserving cognitive domains for the planning and execution of care tasks. Given the characteristics of elderly caregivers, the cognitive health of this population has become an emerging research topic, as well as a public health problem, since a decline in this aspect jeopardizes the caregiver’s ability to provide care for a loved one and perform self-care.^12^

Investigations of the impact of caring on the cognitive performance of caregivers remain controversial and few studies assess elderly caregivers of older adults. Thus, the aim of the present study was to compare cognitive performance and aspects of the care context in elderly caregivers of older adults in a three-year follow-up investigation.

## METHODS

### Participants

A longitudinal study with a three-year follow-up was conducted using data from a survey entitled “Follow-up of elderly caregivers in primary care”, involving elderly caregivers of older adults from a city in the state of São Paulo, Brazil.

The baseline study was conducted from April to November 2014, involving a convenience sample selected from households listed by the 15 Family Health Units, in which two or more older adults resided. The sample consisted of 142 elderly caregivers who met the following inclusion criteria: 60 years of age or older, registered with one of the 15 Family Health Units in the city and being the primary caregiver of a dependent elderly individual living in the same household. Dependence on the part of the elderly care recipient was defined as requiring assistance for at least one activity of daily living (ADL) and/or instrumental activity of daily living (IADL) according to the Katz Index^19^ and the Lawton and Brody Scale.^20^ In this study, a low cognitive test score was not considered an exclusion criterion, but candidates with communication difficulties preventing their understanding of the questions were excluded.

Follow-up data collection was conducted from January to July 2017 and all households were revisited. Twenty-six participants were excluded due to the death of the caregiver, 21 were excluded because the participants had either moved from the city or were not located at their homes after three attempts, 18 were no longer caregivers because the dependent elderly individual had either died or required professional care and 16 refused to participate. Thus, 61 elderly caregivers were submitted to a second evaluation.

### Measures

Data were collected through previously scheduled at-home interviews conducted by trained researchers. The interviews took place in a single session, lasting approximately one hour and thirty minutes.

Caregivers were asked to provide information on sociodemographic characteristics, collected using a questionnaire devised by the researchers, addressing sex (female or male), age (years), education (years), marital status (married, single, divorced or widowed) and ethnicity (white, brown/mulatto or black). Care characteristics were evaluated using a questionnaire inquiring about the relationship between caregiver and care recipient (spouse, father/mother, father-in-law/mother-in-law or brother/sister), time (months) the participant has been providing care, the number of hours/day spent on providing care, whether the participant had received material and/or emotional support (yes/no) and whether the participant had received support from religious groups with regard to providing care (yes/no).

Cognition was assessed using Addenbrooke’s Cognitive Examination-Revised (ACE-R), which is composed of five domains: orientation/attention (18 points), memory (26 points), verbal fluency (14 points), language (26 points) and visuospatial skills (16 points). The total ACE-R score ranges from 0 to 100 points.^21^ In the present study, the ACE-R score was treated as a continuous variable.

Depressive symptoms were assessed using the Geriatric Depression Scale (15-item version), whose final score is determined by summing the item scores.^22^ For this study, a total score ≤5 points was considered indicative of the absence of depressive symptoms, whereas ≥6 points was considered indicative of the presence of depressive symptoms.

Perceived burden was identified using the Brief version of the Zarit Burden Interview (ZBI), composed of 12 items scored on a five-point scale: 0 (not at all) to 4 (always). Total score ranges from 0 to 48 points, with higher scores denoting greater perceived burden^(23,24^ For the analysis, the median of the total score was considered (caregivers with scores above and below the median were considered as having high and low burden, respectively).

### Ethics

This study received approval from the Human Research Ethics Committee of the Federal University of São Carlos (certificate number: 80458017.7.0000.5504). Participation was voluntary and all participants signed a statement of informed consent.

### Statistical analysis

The data were analyzed with the aid of the Statistical Analysis System (SAS) for Windows, version 9.2. Descriptive statistics were performed. Categorical variables were expressed as absolute and percentage frequencies, while continuous variables were expressed as mean and standard deviation values. Pearson’s Chi-square test was used to compare the categorical variables. Due to the non-normal distribution between the two measurements, the Mann-Whitney test was used for the continuous variables. McNemar’s test was used to compare the numerical variables between measurements. The Wilcoxon test for related samples was used due to the absence of a normal distribution. A 5% significance level was adopted for the statistical tests (p≤0.05).

## RESULTS

At the 2014 evaluation, most elderly caregivers were female (n=52; 85.2%), with a mean age of 67.85 years (±5.41), married (n=52; 85.2%) and retirees (n=39; 63.9%). Mean educational level was 3.72 years (±3.4), with a predominance of elementary school education (n=48; 78.7%). The majority of caregivers provided care to a spouse (n=52; 85.2%). Table 1 displays the data on cognitive performance and care variables at the first and second evaluations.

**Table 1:** Cognitive performance and variables of care context among elderly caregivers at both evaluations (n=61), São Carlos-SP, Brazil, 2014-2017.

Variable	Baseline	Follow-up	p-value*
**Cognitive performance**			
MMSE	22.61 (±4.13)	23.03 (±3.61)	0.257*
ACE-R	63.05 (±17.93)	62.30(±16.16)	0.585*
Orientation/attention	13.31 (±2.66)	13.49 (±2.35)	0.348*
Memory	14.93 (±5.97)	14.33 (±5.46)	0.427*
Verbal fluency	5.66 (±2.89)	5.97 (±2.88)	0.111*
Language	17.84 (±5.22)	18.57 (±4.75)	0.024*
Visuo-spatial	22.61 (±4.13)	23.03 (±3.61)	0.257*
**Care context**			
Time providing care (months). Mean (SD)	11.69 (±13.17)	14.79 (±16.69)	0.045**
Time spent on care (hours/day). Mean (SD)	5.10 (±4.34)	6.12 (±5.18)	0.258**
Dependence of care recipient (Katz)			
• Independent n (%)	47 (77.0%)	40 (65.6%)	0.042***
• Dependent n (%)	14 (23.0%)	21 (34.4%)	
Dependence of recipient (Lawton)			
• Partial dependency. n (%)	55 (90.1%)	57 (93.4%)	0.157***
• Total dependency. n (%)	6 (9.9%)	4 (6.6%)	
Receives material support			
• No. n (%)	55 (90.1%)	48 (78.7%)	0.020****
• Yes. n (%)	6 (9.9%)	13 (21.3%)	
Receives emotional support			
• No. n (%)	31 (50.8%)	32 (52.5%)	0.853****
• Yes. n (%)	30 (49.2%)	29 (47.5%)	
• ZBI. Mean(SD)	7.18 (±8.01)	8.46 (±8.80)	0.193*
• ZBI >median. n (%)	34 (55.7%)	32 (52.5%)	0.670***
• ZBI <median. n (%)	27 (44.3%)	29 (47.5%)	
• GDS total. Mean (SD)	4.08 (±2.70)	3.68 (±2.68)	0.250*
• No symptoms. n (%)	45 (75.0%)	47 (78.3%)	0.617***
• With symptoms. n (%)	15 (25.0%)	13 (21.7%)	

* Wilcoxon test;

** Mann-Whitney test;

*** McNemar's test;

**** Pearson's Chi-square test; MMSE: Mini-Mental State Examination; ACE-R: Addenbrooke's Cognitive Examination-Revised; ZBI: Zarit Burden Interview; GDS: Geriatric Depression Scale; SD: standard deviation.

On the cognitive test, a significant increase in the language domain was found at the follow-up evaluation compared to baseline (p=0.024). Regarding the care variables, significant increases between the first and second evaluations were found for receiving material/financial support (p=0.020), time providing care (p=0.045) and the dependence of the elderly care recipient for activities of daily living (p=0.042) (Table 1).

## DISCUSSION

The socio-demographic characteristics of the elderly caregivers in the present investigation are similar to those described in national and international studies, with a predominance of married, retired women with low educational level who provide care for a spouse.^4^^,^ 5 ^,^ 25

Regarding cognitive performance, the elderly caregivers showed an increase between baseline and final evaluations in scores on the ACE-R domains, although the only significant increase was in the language domain. These findings differ from data described in previous studies, which report that caregivers, especially caregivers of an individual with dementia, experience a decline in cognitive performance.^15^^,^ 18

However, tasks linked to caring can be cognitively complex, requiring attention, judgment and problem-solving skills,^11^ which may exert a positive influence on the cognitive functioning of informal caregivers. Indeed, a population-based study involving elderly caregivers found that informal care may be beneficial to cognitive function, especially for female caregivers.^12^ Providing care is an activity that requires adequate cognitive processing and strong performance regarding cognitive skills, such as attention, memory, planning, and logical reasoning.^13^

Informal care can involve everything from physical tasks to psychological activities, which differ in terms of cognitive involvement and intensity. Therefore, the effects on cognition may vary according to the type of care provided.^12^ Psychological well-being is a central factor in cognitive performance, where caregivers can present with a cognitive deficit in specific functions, sometimes associated with burden, stress or depressive symptoms.^6^^,^ 13 ^,^ 26 ^,^ 27 In the present study, the elderly caregivers exhibited low levels of care-related burden and depressive symptoms. A review of population-based studies involving informal caregivers found that low levels of care-related stress are associated with the benefits of caring.^28^

Over the years, the age of older care recipients has increased and probably need more care, increasing the time spent on activities of daily living. The intensity of care can be a source of health effects of care. Studies have compared medium or moderate caregivers with intensive caregivers based on care hours and found greater health effects when more intensive care is provided.^29^^,^ 30

A higher level of dependence of the elderly care recipient results in more time spent on care by the caregiver, reducing the time available for their other activities. The reorganization of time use by family caregivers and the provision of formal support can reduce the burden of caring and benefit the well-being of caregivers.^31^

In the present study, elderly caregivers reported receiving more material/financial support at follow-up compared to baseline. This finding may be related to the increasing age of older care recipients, which may increase dependence on self-care or financially. Receiving support can provide a positive view of life and stressful situations, thereby ensuring the ability to cope with adversity.^32^^,^ 33 Even extreme situations, such as an excessive care burden, can be minimized by social support and coping mechanisms.^33^^,^ 34

Support networks, material/emotional support and social activities are associated with better cognitive function and a lower risk of cognitive decline.^35^ Some studies have demonstrated the positive effects of support on psychological and physical wellbeing in caregivers.^36^^,^ 37

The present investigation has limitations that should be taken into account. The elderly caregivers were evaluated at two discrete timepoints, with no monitoring of the cognitive, psychological and health changes that may have occurred during the time interval between the two evaluations. We therefore suggest that future studies monitor these aspects more regularly. Another limitation involves the loss of individuals between evaluations, making it impossible to determine whether the elderly caregivers who were not reassessed improved or worsened for the aspects evaluated.

The present findings have clinical implications for care provision by older adults to other older adults. Caregivers may be somewhat younger and have greater functional capacity compared to care recipients, but special attention should be given to those who feel burdened by the care. This situation can compromise wellbeing of the caregiver, as well as the quality of the care offered.

In conclusion, the present study, at the follow-up evaluation the elderly caregivers had better performance on the language domain of cognition, spent more time on providing care, elderly care recipients were more dependent for basic activities of daily living, and caregivers received more material/financial support.

The present findings can contribute to the planning and implementation of interventions for elderly caregivers. The role of primary care services is to promote health and prevent disease. Thus, interventions aimed at stimulating physical and psychological health can improve the quality of life of older caregivers and the quality of care provided to older people.
